# Simple analytical method for determining electrical resistivity and sheet resistance using the van der Pauw procedure

**DOI:** 10.1038/s41598-020-72097-1

**Published:** 2020-10-02

**Authors:** F. S. Oliveira, R. B. Cipriano, F. T. da Silva, E. C. Romão, C. A. M. dos Santos

**Affiliations:** Escola de Engenharia de Lorena, University of São Paulo, 12.602-810, Lorena, SP Brazil

**Keywords:** Physics, Techniques and instrumentation, Characterization and analytical techniques, Materials science, Condensed-matter physics, Electronic properties and materials

## Abstract

This work reports an analytical method for determining electrical resistivity (*ρ*) and sheet resistance (*R*_*S*_) of isotropic conductors. The method is compared with previous numerical solutions and available experimental data showing a universal behavior for isotropic conductors. An approximated solution is also reported allowing one to easily determine *ρ* and *R*_*S*_ for samples either with regular or arbitrary shapes.

## Introduction

The study of electrical transport properties is of great importance since is routinely used in many theoretical and experimental researches as well as in many applications. They are especially important for characterizing many materials such as isotropic and anisotropic polycrystalline samples, metal plates, single crystals in bulk and plate-like forms^1–4^, homogeneous and heterogeneous thin films^5–7^, 2D electrical conductivity in exfoliated materials such as graphene and MoS_2_^8–10^, conventional and non-conventional superconductors^11–15^, organic conductors and superconductors^5,11^, Si-based compounds for electronic applications^16,17^, new materials for energy storage such as modern batteries, fuel cells, and solar panels^18–20^, and even for biological specimens^21,22^. Furthermore, the study of phase transitions^23,24^, structural properties^25,26^, and anisotropic properties^1,27^ have been important for materials science and engineering applications. With regard to the physical properties, measuring electrical conductivity is of great relevance for understanding several physical phenomena such as superconductivity^14,15,28,29^, topological materials^9,13,30,31^, 1D and 2D conductivity^3,10,11,28^, quantum Hall effect^10,32^, electronic and quantum phase transitions^3,12,23,33^, and many other physical effects. In many of these applications one of the broadest interests is dealing with samples of irregular shapes and small sizes.

In this particular work, we report a simple analytical procedure to determine electrical resistivity and sheet resistance of isotropic conductors either with regular or arbitrary shapes.

**Figure 1:**
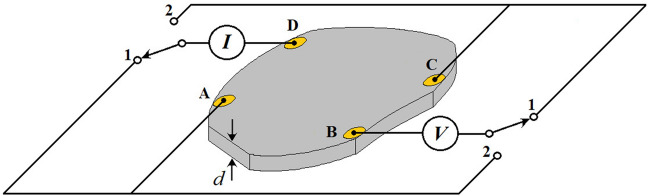
Schematic of a sample with thickness *d* and electrical contacts prepared using the van der Pauw procedure. Electrical resistances *R*_1_ = *R*_*BC,DA*_ = *V*_*BC*_/*I*_*DA*_ and *R*_2_ = *R*_*AB,CD*_ = *V*_*AB*_/*I*_*CD*_ are measured by switching the contacts from position 1 to 2, respectively. Thermoelectric voltages associated with the electrical contacts to the sample are eliminated by measuring each dc voltage for both polarities in the following way: *R*_1_ = *R*_*BC,DA*_ = (*V*^+^_*BC*_ – *V*^*-*^_*CB*_*)*/(*I*^+^_*DA*_ – *I*^*-*^_*AD*_) and *R*_2_ = *R*_*AB,CD*_ = (*V*^+^_*AB*_ – *V*^*-*^_*BA*_*)*/(*I*^+^_*CD*_ – *I*^*-*^_*DC*_). This figure was adapted from Karls and Hoch^36^. License: 00331-20020-00000-AA293, Paint/Windows 10, www.microsoft.com/pt-br/windows.

One of the procedures used to determine of electrical resistivity and sheet resistance of conducting materials with regular or arbitrary shape was proposed more than 60 years ago by van der Pauw (vdP)^34,35^. In this procedure four electrical contacts are placed on the sample, as shown in Fig. 1. Current and voltage contacts are cycled through switches 1 and 2.

Based upon the measured *R*_1_ and *R*_2_ values, electrical resistivity (*ρ*) and sheet resistance (*R*_*S*_ = *ρ*/*d*) can be determined by^34,35^

1 $$\rho = \frac{\pi d}{\mathrm{ln}2}\left(\frac{{R}_{1}+{R}_{2}}{2}\right)f\left(\frac{{R}_{2}}{{R}_{1}}\right),$$

where *d* is the thickness of the sample and *f*(*R*_2_*/R*_1_) is a geometric factor that is, up to now, determined numerically or graphically. Since the vdP method was introduced^34^ others have suggested various ways to determine *f(R*_2_*/R*_1_*)*. Ramadan et al*.*^37^ provided values of *f*(*R*_2_*/R*_1_) for 1 ≤ *R*_2_*/R*_1_ ≤ 200. de Vries and Vieck^38^ reported some results for *f*(*R*_2_*/R*_1_) using polyethylene samples with parallelepiped shapes with aspect ratio (length/width) between approximately 0.6 and 2.6. At present, *f*(*R*_2_*/R*_1_) is the best determined using the method of Chan^39^ who solved numerically the transcendental equation

2 $$\frac{{R}_{2}-{R}_{1}}{{R}_{2}+{R}_{1}}\frac{\mathrm{ln}2}{f}={\mathrm{acosh}}\left[\frac{exp(\frac{\mathrm{ln}2}{f})}{2}\right],$$

for 1 ≤ *R*_2_*/R*_1_ ≤ 10^4^ with high precision.

In addition, some authors have discussed non-ideal conditions in the vdP measurements taking into account corrections due to contacts, sample thickness, and sample inhomogeneity^36,40–44^. In this particular work, the samples are considered isotropic, homogeneous in composition and in thickness, with electrical contacts infinitely small compared to size of the samples, placed at the border of the them.

## Results and discussion

Montgomery^45^, using a similar procedure as illustrated in Fig. 1, but for parallelepiped samples, developed a graphical method to determine the electrical resistivity of both isotropic and anisotropic samples, which was supported by the calculation of the electrostatic potential for rectangular blocks reported by Logan et al*.*^46^ and the Wasscher transformation from isotropic to anisotropic sample^47^. A modification of the Montgomery method^45^ has allowed us to determine the electrical resistivity and sheet resistance of parallelepiped samples based upon simple equations^48^.

**Figure 2:**
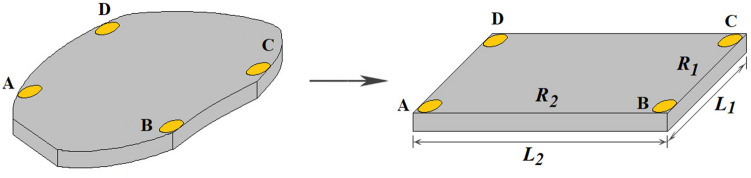
Conformal transformation of a sample with arbitrary shape in the van der Pauw method into parallelepiped sample in the Montgomery method. The equivalent parallelepiped sample, with same thickness *d* and electrical resistances *R*_1_ and *R*_2_ of the real sample, has sides *L*_1_ and *L*_2_. The *L*_2_/*L*_1_ ratio is calculated based upon *H*_1_/*H*_2_ = *R*_2_/*R*_1_, where *R*_1_ and *R*_2_ are determined experimentally, and *H*_1_ and *H*_2_ are given by Eqs. (4) and (5). License: 00331-20020-00000-AA293, Paint/Windows 10, www.microsoft.com/pt-br/windows.

Using the Montgomery method^45,47^ one can find electrical resistivity of isotropic parallelepiped samples by

3 $$\rho ={H}_{1}E{R}_{1}={H}_{2}E{R}_{2},$$

where *E* is the effective thickness, which measures the penetration depth of the electrical current into the sample; it is equal to *d* in Fig. 1 for thin samples *H*_1_ and *H*_2_ are functions of *L*_2_/*L*_1_ given by^46,48^

4 $$\frac{1}{{H}_{1}}=\frac{8}{\pi }\sum_{n=0}^{\infty }\frac{1}{\left(2n+1\right){\mathrm{sinh}}\left[\pi \left(2n+1\right){L}_{2}/{L}_{1}\right]}$$

and

5 $$\frac{1}{{H}_{2}}=\frac{8}{\pi }\sum_{n=0}^{\infty }\frac{1}{\left(2n+1\right){\mathrm{sinh}}\left[\pi \left(2n+1\right){L}_{1}/{L}_{2}\right]},$$

where *L*_1_ and *L*_2_ are the length and width of a particular parallelepiped sample^46,48^ (see Fig. 2).

Despite the shape of the samples, Eqs. (1) and (3) that must be equal for a given conducting material. This implies

6 $$f\left({H}_{1},{H}_{2}\right)=\frac{2\mathrm{ln}2}{\pi }\left(\frac{1}{1+{R}_{2}/{R}_{1}}\right){H}_{1}=\frac{2\mathrm{ln}2}{\pi }\left(\frac{1}{1+{R}_{1}/{R}_{2}}\right){H}_{2},$$

for thin samples (*E* = *d*). Furthermore, using Eq. (3) again to eliminate *R*_*2*_/*R*_*1*_, one can obtain

7 $$f\left({H}_{1},{H}_{2}\right)=\frac{2\mathrm{ln}2}{\pi }{\left(\frac{1}{{H}_{1}}+\frac{1}{{H}_{2}}\right)}^{-1},$$

which after combining with Eq. (2) and making some algebraic manipulations (see Supplementary material) leads to

8 $${e}^{-\frac{\pi }{{H}_{2}}}+{e}^{-\frac{\pi }{{H}_{1}}}=1.$$

**Figure 3:**
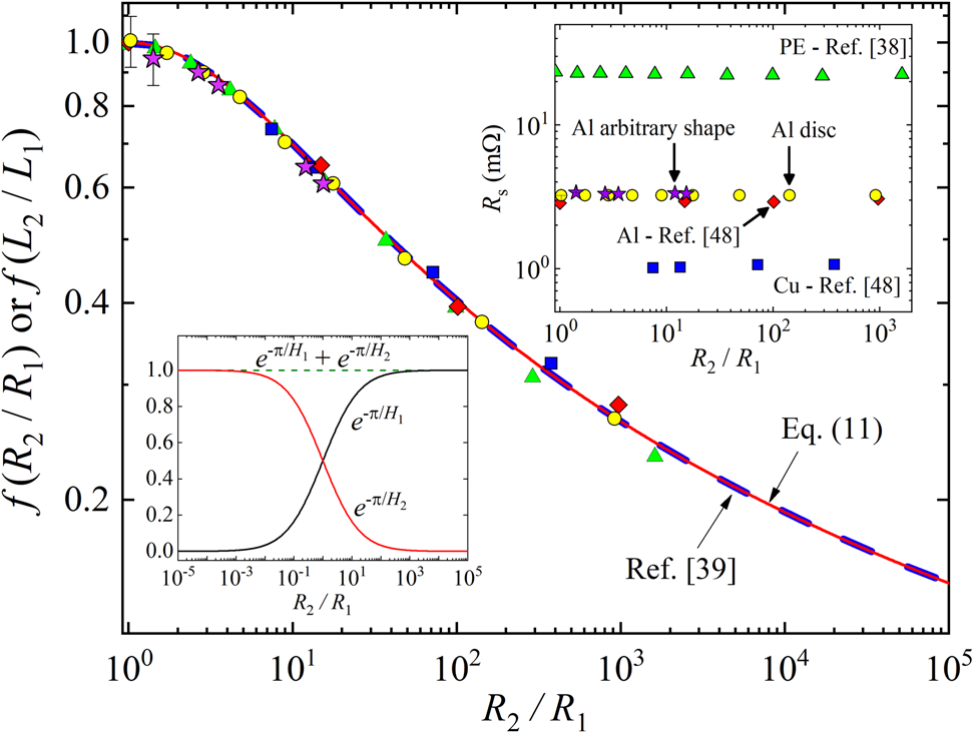
Geometric factor *f* (*R*_2_/*R*_1_) and *f* (*L*_2_/*L*_1_) in the van der Pauw method versus *R*_2_*/R*_1_. Red line represents the behavior predicted by Eq. (11) with *H*_*1*_ calculated using Eq. (4) with the procedure described in section II of the Supplementary material. The red curve overlaps to the numerical solution by Chan^39^, displayed by the blue dashed line. Symbols represent data from this work and published values^38,48^. Geometric factor is shown only for *R*_2_/*R*_1_ ≥ 1 because it is symmetric with regard to *R*_2_/*R*_1_ ≤ 1. Uncertainties shown in *f*(*R*_2_/*R*_1_) for two points near *f* = 1 were calculated using Eq. (1) by propagating the uncertainties due to *R*_*S*_ determined by standard four probe method (see the calculation for the circle and star symbols in Tables 2 and 3). Same proportional uncertainties apply to the yellow and pink symbols. Lower inset demonstrates that *H*_1_ and *H*_2_ provide an exact solution for the transcendental equations by vdP. Upper inset displays *R*_*S*_ (= *ρ*/*d*) for five different experimental set of measurements calculated with Eq. (12). The constant values of *R*_*S*_ show how precise the procedure reported here is over the typical *R*_2_/*R*_1_ range of measurements, 1 ≤ *R*_2_/*R*_1_ ≤ 10^3^. License: GF3S5-3078-7903112, Origin 2018 (9.5), www.originlab.com.

This result recovers the fundamental equation of van der Pauw method^34,35^

9 $${e} ^ { \left( - \frac{\pi {R}_{2}E}{\rho}\right)} +{e}^{\left( -\frac{\pi {R}_{1}E}{\rho}\right)}=1,$$

which shows that *f*(*H*_1_*,H*_2_) given by Eq. (7) is exact solution of the transcendental Eq. (2). A confirmation can be observed in the lower inset of Fig. 3, which shows that adding the two terms on the left side of the Eq. (8) yields unity, no matter the *R*_2_*/R*_1_ ratio.

Additionaly, from Eq. (8), one can write *H*_2_ as a function of *H*_1_, as

10 $${H}_{2}=-\frac{\pi }{{\mathrm{ln}}\left(1-{e}^{-\frac{\pi }{{H}_{1}}}\right),}$$

which can be used to eliminate *H*_2_ in Eq. (7), yielding the geometric factor

11 $$f\left(\frac{{L}_{2}}{{L}_{1}}\right)=2{\mathrm{ln}}2 \, \left(\frac{1}{\pi /{H}_{1}-{\mathrm{ln}}(1-{e}^{-\pi /{H}_{1}}) \, \, }\right),$$

since *H*_1_ is only a function of *L*_2_/*L*_1_, given by Eq. (4). Eliminating *H*_2_ in Eq. (7) is of special interest because the convergence of 1/*H*_1_ series is much faster than 1/*H*_2_ (see Supplementary material).

Thus, electrical resistivity and sheet resistance can be exactly determined by

12 $$\rho =\pi d\left({R}_{1}+{R}_{2}\right)\left(\frac{1}{\pi /{H}_{1}-{\mathrm{ln}}(1-{e}^{-\pi /{H}_{1}}) \, \, }\right).$$

An important aspect associated with above results is the fact that the equations in the Montgomery method are related to parallelepiped samples, but solve the transcendental equations in the vdP method, which work not only for parallelepiped samples but also for any regular and arbitrary shapes. This is valid because there is a conformal transformation, in a similar way as described by others^34,38,49^ of the isotropic sample, with arbitrary shape and measurements *R*_1_ and *R*_2_ in the vdP method, into an equivalent parallelepiped sample with the same electrical resistances and correspondent sides *L*_1_ and *L*_2_ in the Montgomery method. This idea is displayed in Fig. 2. Once the equivalent parallelepiped sample is defined, equations of the Montgomery method can be used to solve Eqs. (1) and (2) by vdP. Such as *H*_1_ and *H*_2_ are functions of the *L*_2_/*L*_1_ (see Eqs. (4) and (5) again), *f*(*H*_2_*,H*_1_) and *f*(*L*_2_/*L*_1_) given by Eq. (7) or (11) shows that this parameter is indeed a geometric factor, in agreement with previous reports^34,45,46^.

Two cases of this conformal transformation are of particular interest: (i) a sample with parallelepiped shape is its own equivalent sample, and (ii) a non-parallelepiped sample with *R*_*1*_ = *R*_2_ (*f*(1) = 1) is represented by an equivalent square sample. Furthermore, a sample with square shape (*L*_1_ = *L*_2_) has also special interest, since *R*_1_ and *R*_2_ must have same values, no matter the size of the sample (see, for instance, results for Al foils in Table II of the reference^48^). Since *R*_1_ = *R*_2_ for any square sample, *R*_1_ + *R*_2_ must be constant regardless the length of the squares, which is expected because sheet resistance must be the same for a given conducting material with homogeneous thickness (see Eq. (1) again).

In order to understand this transformation better, we compare the *f*(*L*_2_*/L*_1_) from the exact solution given by Eq. (11) with *f*(*R*_2_*/R*_1_) from different numerical calculations^34,35,37–39^. Figure 3 displays the high precision numerical solution reported by Chan^39^ and the expected behavior for Eq. (11) (see also Supplementary material). Available experimental data reported previously^38,48^ and measured in this work are also shown in Fig. 3.

**Figure 4:**
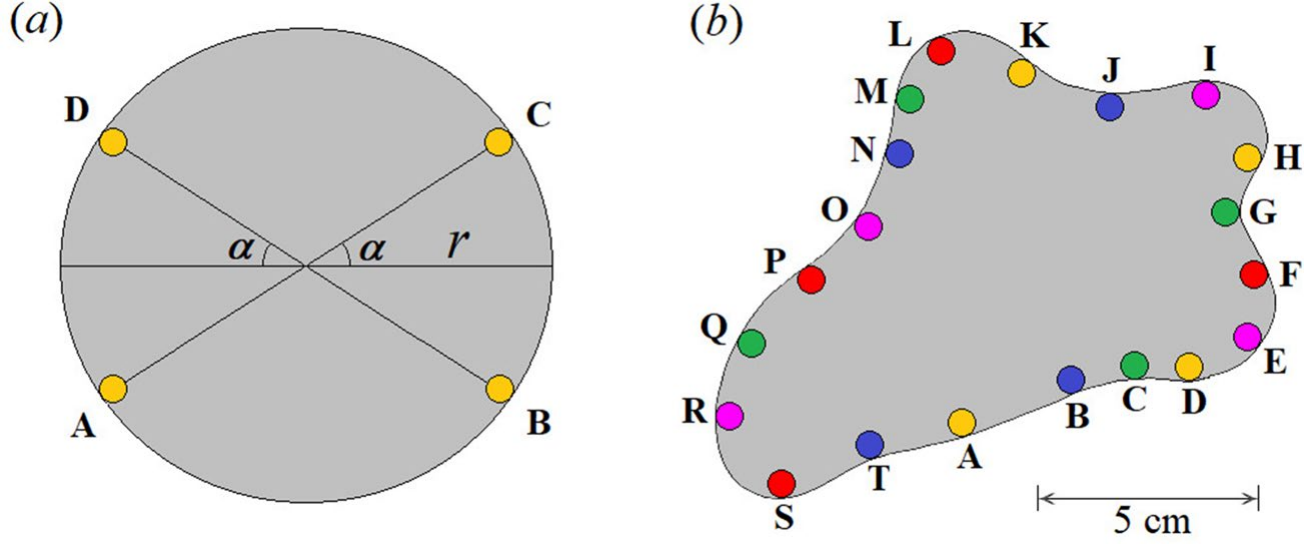
Shape of the samples measured in this work. They were made out from Al foil with **(a)** circular shape (*r* = 5 cm) and **(b)** arbitrary shape, as used by vdP^34^. Positions indicated by letters represent the positions of the electrical contacts for the measurement data shown in Tables 2 and 3. License: 00331-20020-00000-AA293, Paint/Windows 10, https://www.microsoft.com/pt-br/windows.

In Fig. 3, one can see a clear the overlap between the expected behavior by Eq. (11), indicated by red line, and the numerical calculation by Chan^39^ shown by the blue dashed line. Overlap (not shown) with other results reported previously were also observed^34,35,37,38^. Additionally, symbols shown in Fig. 3 are related to some experimental data available in literature for polyethylene (PE) samples (green triangles)^38^, Cu metal sheet plates (blue squares), and Al foil (red diamonds)^48^. Yellow circles and pink stars are due to measurements with samples made out of Al foil with a circular and arbitrary shape, respectively, as displayed in Fig. 4. In order to test the transformation proposed in this work, we measured a sample with arbitrary shape, as used by van der Pauw (see Fig. 3 in Ref.^34^).

The electrical resistivity used to determine *f*(*R*_2_*/R*_1_) for the experimental data plotted in Fig. 3 was measured using standard four-probe method, measuring square samples with Montgomery method^45^, or searching for *R*_1_ = *R*_2_ during the application of the vdP method, in which *f*(1) = 1 (equivalent square sample). As shown in Fig. 3, all the data for the several isotropic samples collapse into a universal behavior, despite the shapes and materials.

The upper inset of the Fig. 3 displays the results of the sheet resistance calculated by Eq. (12) for the five experimental sets of data. The results show that *R*_*S*_ is almost constant for each material over the measured range: 1 ≤ *R*_2_*/R*_1_ ≤ 10^3^, as expected.

Regarding practical purposes, as reported previously^48^ *H*_1_ and *H*_2_ can be truncated in the first term of each series, as ${H}_{1}\approx \frac{\pi }{8}{\rm sinh}(\pi G)$ and ${H}_{2}\approx \frac{\pi }{8} {\rm sinh}(\pi /G)$, providing an approximated solution for the geometric factor from Eq. (7) as

13 $$f\left(G\right)\approx \frac{\mathrm{ln}2}{4 \left[{\rm csch}\left(\pi G\right)+{\rm csch} \left(\pi /G\right)\right]},$$

where *G* ≈ *L*_2_/*L*_1_ is a dimensionless geometric parameter for the equivalent parallelepiped sample which can be easily estimated by

14 $$G\approx \frac{1}{2}\left\{\frac{1}{\pi }{\mathrm{ln}}\left(\frac{{R}_{2}}{{R}_{1}}\right)+\sqrt{{\left[\frac{1}{\pi }{\mathrm{ln}}\left(\frac{{R}_{2}}{{R}_{1}}\right)\right]}^{2}+4}\right\},$$

that is a good approximation to find *L*_2_/*L*_1_ from 1 up to 4, corresponding to *R*_2_*/R*_1_ from 1 up to ~ 10^5^.

**Figure 5:**
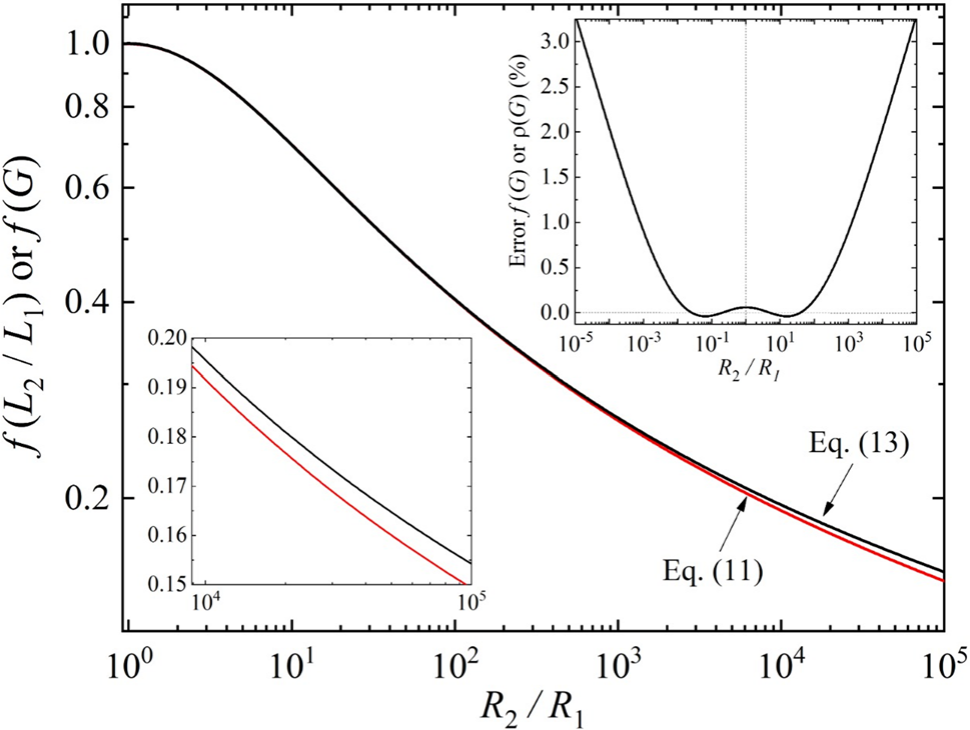
Comparison between exact solution *f*(*L*_2_/*L*_1_) and approximated solution *f*(*G*) given by Eqs. (11) and (13), respectively. In lower inset is displayed a zooming from 10^4^ to 10^5^. Upper inset shows the percentage difference between *f*(*R*_2_/*R*_1_) and *f*(*L*_2_/*L*_1_). Error is less than 3.3% over the 10^–5^ ≤ *R*_2_/*R*_1_ ≤ 10^5^ range. License: GF3S5-3078-7903112, Origin 2018 (9.5), https://www.originlab.com.

Figure 5 displays the behavior of *f*(*G*) given by Eq. (13) and compares with the exact solution of *f*(*L*_2_/*L*_1_) predicted by Eq. (11).

The behavior of the black line in Fig. 5 demonstrates that the Eq. (13) for *f*(*G*), along with (14), is a good approximation to determine *f*(*L*_2_/*L*_1_), which is very close to the red line. The error of *f*(*R*_2_/*R*_1_) shown in inset of the Fig. 4 is less than 3.3% over the 10^–5^ ≤ *R*_2_/*R*_1_ ≤ 10^5^ range. For the practical range of *R*_*1*_ and *R*_*2*_ measurements (typically *R*_2_/*R*_1_ ≤ 10^3^) the error is less than 1%. Thus, the benefits of using the approximated solution can be considered excellent since the uncertainties due to the size of the electrical contacts during samples preparation are typically much higher. A numerical comparison between *f*(*L*_2_/*L*_1_) and *f*(*G*), for selected values in the *R*_2_/*R*_1_ range shown in Fig. 5 is displayed in Table 1.

**Table 1:** Some selected values for the parameters discussed in this work. *L*_2_/*L*_1_, *H*_1_, *H*_2_, and *f*(*L*_2_/*L*_1_) are numerically calculated using Eqs. (4), (10), and (11) with *n* = 0. *G* and *f*(*R*_2_/*R*_1_) are calculated using the approximations given by Eqs. (13) and (14). Error of *f*(*G*) was obtained after comparison with *f*(*L*_*2*_/*L*_*1*_) values. For details of the calculations refer to Section II of the Supplementary material.

*R*_2_/*R*_1_ = *H*_1_/*H*_2_	1	10	10^2^	10^3^	10^4^	10^5^
*L*_2_/*L*_1_	0.99980	1.42802	1.95900	2.55341	3.18450	3.83791
*H*_1_	4.53236	17.4311	92.4347	598.193	4,343.92	33,836.5
*H*_2_	4.53236	1.74311	0.92435	0.59819	0.43439	0.33837
*f*(*L*_2_/*L*_1_)	1.00000	0.69926	0.40385	0.26370	0.19167	0.14931
*G*	1.00000	1.43150	1.97277	2.58557	3.24035	3.91979
*f*(*G*)	1.00062	0.69906	0.40443	0.26608	0.19557	0.15424
Error *f*(*G*) (%)	− 0.062	− 0.029	0.144	0.902	2.035	3.302

Based upon above discussion, one can rewrite Eq. (1) as

15 $$\rho\left(G\right) =\frac{\pi d}{8 }{(R}_{1}+{R}_{2})\frac{1}{\left[{\rm csch}\left(\pi G\right)+{\rm csch}\left(\pi /G\right)\right]},$$

which is a simple approximated function that allows the determination of *R*_*S*_ or *ρ* for thin samples (*E* = *d*) directly from the *R*_1_ and *R*_2_ measurements. If *R*_1_ and *R*_2_ are measured with high precision, the error of *ρ* in Eq. (15) is the same as the *f*(*G*), which is displayed in upper inset of the Fig. 5 and Table 1.

Finally, the conformal transformation described above is verified analyzing the measurements of the samples displayed in Fig. 4, with the method reported here. Table 2 and 3 provided the results for both samples, whose different electrical contacts were used to measure *R*_1_ and *R*_2_, as indicated in the corresponding tables.

**Table 2:** Experimental data set measured using the sample with the circular shape shown in Fig. 4a.

α (degree)	*R*_2_ (mΩ)	*R*_1_ (mΩ)	*R*_2_/*R*_1_	*f*(*R*_2_/*R*_1_)	*R*_*S*_ (mΩ)	*G*	*f*(*L*_2_/*L*_1_)	*R*_*S*_ (*G*) (mΩ)
5	5.349(3)	0.0058(8)	920(120)	0.26(2)	3.243	2.562	0.270	3.273
10	3.786(1)	0.027(1)	140(6)	0.37(3)	3.237	2.064	0.376	3.246
15	2.988(1)	0.0619(9)	48.3(7)	0.46(4)	3.286	1.792	0.476	3.287
20	2.217(1)	0.1255(6)	17.7(1)	0.60(6)	3.220	1.556	0.607	3.221
25	1.821(1)	0.2028(1)	8.98(4)	0.70(7)	3.293	1.409	0.718	3.292
30	1.428(1)	0.3002(7)	4.76(2)	0.82(8)	3.251	1.279	0.831	3.252
35	1.174(1)	0.4107(8)	2.859(9)	0.89(8)	3.282	1.181	0.914	3.283
40	0.934(1)	0.545(1)	1.714(5)	0.95(9)	3.269	1.090	0.976	3.271
45	0.718(1)	0.699(1)	1.026(3)	1.00(9)	3.210	1.004	1.001	3.212

α defines the position of the electrical contacts A to D. *f*(*R*_2_/*R*_1_) is calculated using Eq. (1) with *R*_*S (4P)*_ determined through conventional four probe measurement. *R*_*S*_ is obtained using Eq. (12), after determining *H*_*1*_ in a similar manner as described in Table 1. Values of *G*, *f*(*G*), and *R*_*S*_(*G*) are calculated using Eqs. (13) to (15).

**Table 3:** Experimental data for a sample with arbitrary shape shown in the Fig. 4b.

	*R*_2_ (mΩ)		*R*_1_ (mΩ)		*R*_2_/*R*_1_	*f*(*R*_2_/*R*_1_)	*G*	*R*_*S*_(*G*) (mΩ)
	AK, DH	0.883(2)	AD, KH	0.626(2)	1.411(8)	0.94(9)	1.056	3.387
	TN, BJ	1.1501(3)	TB, NJ	0.4332(5)	2.655(4)	0.89(8)	1.167	3.319
	QC, MG	1.2925(6)	QM, CG	0.3665(3)	3.527(5)	0.85(8)	1.221	3.314
	OI, RE	2.039(2)	RO, EI	0.1699(6)	12.00(5)	0.64(6)	1.471	3.345
	SF, PL	2.2012(4)	SP, FL	0.1431(4)	15.38(5)	0.60(6)	1.526	3.337

Letters and the colors in next to *R*_1_ and *R*_2_ values represent the positions of the electrical contacts. *f*(*R*_2_/*R*_1_), *G*, and *R*_*S*_ were calculated using Eqs. (1), (14), and (15), respectively. Uncertainties in *R*_1_ and *R*_2_ are determined by the linear regression of IV curves and uncertainties in *f*(*R*_2_/*R*_1_) are determined by propagating the error due to *R*_*S* (4P)_ determined by standard four probe method.

Results in Table 2 and 3 show that the Eqs. (13) to (15) allow one to determine easily the sheet resistance of the aluminum foil in both samples. All values of the sheet resistances are close to each other and the averages, < *R*_*S*_ >  = 3.25(3) and < *R*_*S*_(*G*) >  = 3.26(3) mΩ for the circular sample, and < *R*_*S*_(*G*) >  = 3.34(3) mΩ for the sample with arbitrary shape, agree very well, within of uncertainty, with regard to the value determined from standard four-probe method, *R*_*S* (4P)_ = 3.2(3) mΩ. Especially interesting is the result in the first line of the Table 3, related to the shape and the electrical contacts of the sample that is similar to the one reported by vdP^34^ (yellow contacts in Fig. 4b). In such a case, R_1_ and *R*_2_ measurements are similar and yield *f*(*R*_2_*/R*_1_) ~ 1, suggesting that the electrical contacts in the historical sample by vdP^34^ has to do with an equivalent square sample.

## Conclusion

In summary, a simple method for determining electrical resistivity and sheet resistance of isotropic samples has been obtained by comparing electrical resistivity equations given by vdP and Montgomery methods. The transcendental equation reported in the vdP method has been solved both exactly and approximately by Eqs. (12) and (15), respectively. The geometric factor, regardless of the shape of the sample, can be determined based upon *H*_1_ and *H*_2_ series, which depend on *L*_2_/*L*_1_, the ratio between the length and width of the equivalent parallelepiped sample. A comparison of the geometric factor with previous numerical calculations and experimental data shows a universal behavior for any isotropic conducting materials. The method reported can be used for measuring electrical properties of many materials, as noted in the introduction. Compared with the best numerical calculation reported by Chan^39^, the method described here is much simpler to use. As far as we know, this is the first time since the report for the vdP method^34^ more than 60 years ago, that an analytical solution for this method has been reported.

## Supplementary information

Supplementary Information.

## References

[1] Borup KA, Fischer KFF, Brown DR, Snyder GJ, Iversen BB (2015). Measuring anisotropic resistivity of single crystals using the van der Pauw technique. Phys. Rev. B.

[2] Goble NJ (2017). Anisotropic electrical resistance in mesoscopic LaAlO_3_ /SrTiO_3_ devices with individual domain walls. Sci. Rep..

[3] Ichinokura S (2019). Vortex-induced quantum metallicity in the mono-unit-layer superconductor NbSe_2_. Phys. Rev. B.

[4] Kim JY (2014). Abnormal drop in electrical resistivity with impurity doping of single-crystal Ag. Sci. Rep..

[5] Rolin C (2017). Charge carrier mobility in thin films of organic semiconductors by the gated van der Pauw method. Nat. Commun..

[6] Flatten, T. *et al.* Direct measurement of anisotropic conductivity in a nanolaminated (Mn_0.5_Cr_0.5_)_2_GaC thin film. *Appl. Phys. Lett.* **115**, 094101 (2019).

[7] Dutta S (2017). Thickness dependence of the resistivity of platinum-group metal thin films. J. Appl. Phys..

[8] Lee GH, Park GH, Lee HJ (2015). Observation of negative refraction of Dirac fermions in graphene. Nat. Phys..

[9] Qing F (2018). A general and simple method for evaluating the electrical transport performance of graphene by the van der Pauw-Hall measurement. Sci. Bull..

[10] Yin J (2019). Dimensional reduction, quantum Hall effect and layer parity in graphite films. Nat. Phys..

[11] Jerome D, Yonezawa S (2016). Novel superconducting phenomena in quasi-one-dimensional Bechgaard salts. C. R. Phys..

[12] Kuo H-H, Chu J-H, Palmstrom JC, Kivelson SA, Fisher IR (2016). Ubiquitous signatures of nematic quantum criticality in optimally doped Fe-based superconductors. Science.

[13] Balakrishnan G, Bawden L, Cavendish S, Lees MR (2013). Superconducting properties of the in-substituted topological crystalline insulator SnTe. Phys. Rev. B.

[14] Jung S-G (2015). Enhanced critical current density in the pressure-induced magnetic state of the high-temperature superconductor FeSe. Sci. Rep..

[15] Zhong R (2018). Evidence for magnetic-field-induced decoupling of superconducting bilayers in La_2-x_Ca_1+x_Cu_2_O_6_. Phys. Rev. B.

[16] Rollo S, Rani D, Olthuis W, Pascual García C (2019). Single step fabrication of silicon resistors on SOI substrate used as thermistors. Sci. Rep..

[17] Hochbaum AI (2008). Enhanced thermoelectric performance of rough silicon nanowires. Nature.

[18] Chen Y, Jiang C, Cho C (2019). Characterization of effective in-plane electrical resistivity of a gas diffusion layer in polymer electrolyte membrane fuel cells through freeze–thaw thermal cycles. Energies.

[19] Cano ZP (2018). Batteries and fuel cells for emerging electric vehicle markets. Nat. Energy.

[20] Zeitouny J, Katz EA, Dollet A, Vossier A (2017). Band gap engineering of multi-junction solar cells: Effects of series resistances and solar concentration. Sci. Rep..

[21] Pesaran B (2018). Investigating large-scale brain dynamics using field potential recordings: Analysis and interpretation. Nat. Neurosci..

[22] Elbohouty M, Wilson MT, Voss LJ, Steyn-Ross DA, Hunt LA (2013). In vitro electrical conductivity of seizing and non-seizing mouse brain slices at 10 kHz. Phys. Med. Biol..

[23] Tao Q (2016). Nonmonotonic anisotropy in charge conduction induced by antiferrodistortive transition in metallic SrTiO_3_. Phys. Rev. B.

[24] Hsieh SH (2017). Anisotropy in the thermal hysteresis of resistivity and charge density wave nature of single crystal SrFeO_3-δ_: X-ray absorption and photoemission studies. Sci. Rep..

[25] Peng L, Wells SA, Ryder CR, Hersam MC, Grayson M (2018). All-electrical determination of crystal orientation in anisotropic two-dimensional materials. Phys. Rev. Lett..

[26] Pardoen T (2016). A versatile lab-on-chip test platform to characterize elementary deformation mechanisms and electromechanical couplings in nanoscopic objects. C. R. Phys..

[27] Miccoli I, Edler F, Pfnür H, Tegenkamp C (2015). The 100^th^ anniversary of the four-point probe technique: The role of probe geometries in isotropic and anisotropic systems. J. Phys. Condens. Matter.

[28] da Luz, M. S., dos Santos, C. A. M., Moreno, J., White, B. D. & Neumeier, J. J. Anisotropic electrical resistivity of quasi-one-dimensional Li_0.9_Mo_6_O_17_ determined by the Montgomery method. *Phys. Rev. B* **76**, 233105 (2007).

[29] Barišić N (2013). Universal sheet resistance and revised phase diagram of the cuprate high-temperature superconductors. Proc. Natl. Acad. Sci. USA.

[30] Tanaka Y (2012). Experimental realization of a topological crystalline insulator in SnTe. Nat. Phys..

[31] Hung, T. Y. T., Camsari, K. Y., Zhang, S., Upadhyaya, P. & Chen, Z. Direct observation of valley-coupled topological current in MoS_2_. *Sci. Adv.* **5**, eaau6478 (2019). 10.1126/sciadv.aau6478 PMC6474770 31016236

[32] Jeckelmann B, Jeanneret B (2003). The quantum Hall effect as an electrical resistance standard. Meas. Sci. Technol..

[33] Orgiani P (2007). Direct measurement of sheet resistance R□ in cuprate systems: Evidence of a fermionic scenario in a metal-insulator transition. Phys. Rev. Lett..

[34] van der Pauw LJ (1958). A method of measuring specific resistivity and Hall effect of discs of arbitrary shape. Philips Res. Rep..

[35] Pauw, L. J. van der. A. Method of measuring the resistivity and hall coefficient on lamellae of arbitrary shape. *Philips Res. Rep.* **20,** 220–224 (1958/1959).

[36] Kasl C, Hoch MJR (2005). Effects of sample thickness on the van der Pauw technique for resistivity measurements. Rev. Sci. Instrum..

[37] Ramadan AA, Gould RD, Ashour A (1994). On the Van der Pauw method of resistivity measurements. Thin Solid Films.

[38] de Vries DK, Wieck AD (1995). Potential distribution in the van der Pauw technique. Am. J. Phys..

[39] Chan WK (2000). On the calculation of the geometric factor in a van der Pauw sheet resistance measurement. Rev. Sci. Instrum..

[40] Smith BJ, Chwang R, Crowell CR (1974). Contact size effects on the van der Pauw method for resistivity and Hall coefficient measurement. Solid-State Electron..

[41] Koon DW (1989). Effect of contact size and placement, and of resistive inhomogeneities on van der Pauw measurements. Rev. Sci. Instrum..

[42] Wu B (2010). Finite element analysis of the effect of electrodes placement on accurate resistivity measurement in a diamond anvil cell with van der Pauw technique. J. Appl. Phys..

[43] Matsumura T, Sato Y (2010). A theoretical study on van der Pauw measurement values of inhomogeneous compound semiconductor thin films. J. Mod. Phys..

[44] Reveil M, Sorg VC, Cheng ER, Ezzyat T, Clancy P, Thompson MO (2017). Finite element and analytical solutions for van der Pauw and four-point probe correction factors when multiple non-ideal measurement conditions coexist. Rev. Sci. Instrum.

[45] Montgomery HC (1971). Method for measuring electrical resistivity of anisotropic materials. J. Appl. Phys..

[46] Logan BF, Rice SO, Wick RF (1971). Series for computing current flow in a rectangular block. J. Appl. Phys..

[47] Wasscher JD (1961). Note on four-point resistivity measurements on anisotropic conductors. Philips Res. Rep..

[48] dos Santos CAM (2011). Procedure for measuring electrical resistivity of anisotropic materials: A revision of the Montgomery method. J. Appl. Phys..

[49] Lim SHN, McKenzie DR, Van der Bilek MMM (2009). Pauw method for measuring resistivity of a plane sample with distant boundaries. Rev. Sci. Instrum..

