# Supplementary material for: Simple analytical method for determining electrical resistivity and sheet resistance using the van der Pauw procedure
Source: Sci Rep. 2020 Oct 2;10:16379. doi: 10.1038/s41598-020-72097-1 (PMC7532437; doi:10.1038/s41598-020-72097-1)
Supplement: Supplementary file 1 — Supplementary Information. [file 41598_2020_72097_MOESM1_ESM.pdf]

Supplementary material for
Simple Analytical Procedure for Determining Electrical Resistivity and Sheet Resistance Using the van der Pauw Method

F. S. Oliveira, R. B. Cipriano, F. T. da Silva, E. C. Romão, and C. A. M. dos Santos
Escola de Engenharia de Lorena, Universidade de São Paulo, Lorena – SP, 12.602-810, Brazil

I. Proving that $f(L_2/L_1)$ is an analytical solution for the transcendental equations by vdP:

From Eqs. (2) and (3), one can write

$$\frac{H_1-H_2}{H_1+H_2}\frac{\ln 2}{f} = \operatorname{acosh}\left[\frac{exp(\frac{\ln 2}{f})}{2}\right] , \tag{1S}$$

but from Eq. (7)

$$\frac{\ln 2}{f} = \frac{\pi}{2}\left(\frac{1}{H_1}+\frac{1}{H_2}\right) = \frac{\pi}{2}\left(\frac{H_1+H_2}{H_1 H_2}\right) . \tag{2S}$$

Substituting Eq. (2S) into Eq. (1S) yields

$$\frac{\pi}{2}\left(\frac{H_1-H_2}{H_1 H_2}\right) = \operatorname{acosh}\left[\frac{exp\frac{\pi}{2}\left(\frac{1}{H_1}+\frac{1}{H_2}\right)}{2}\right], \tag{3S}$$

or

$$\cosh\left(\frac{\pi}{2}\frac{H_1-H_2}{H_1 H_2}\right) = \frac{1}{2}\left[e^{(\frac{\pi}{2H_1}+\frac{\pi}{2H_2})}\right] . \tag{4S}$$

However,

$$\cosh\left(\frac{\pi}{2}\frac{H_1-H_2}{H_1 H_2}\right) = \frac{1}{2}\left[e^{(\frac{\pi}{2}\frac{H_1-H_2}{H_1 H_2})} + e^{(\frac{\pi}{2}\frac{H_2-H_1}{H_1 H_2})}\right] = \frac{1}{2}\left[e^{(\frac{\pi}{2H_2})}e^{-(\frac{\pi}{2H_1})} + e^{-(\frac{\pi}{2H_2})}e^{(\frac{\pi}{2H_1})}\right] . \tag{5S}$$

Additionally, the right side of the Eq. (3S) provides

$$\frac{1}{2}\left[e^{(\frac{\pi}{2H_1}+\frac{\pi}{2H_2})}\right] = \frac{1}{2}e^{\frac{\pi}{2H_1}}e^{\frac{\pi}{2H_2}} , \tag{6S}$$

which means from (4S) that

$$e^{-\frac{\pi}{2H_1}}e^{\frac{\pi}{2H_2}} + e^{\frac{\pi}{2H_1}}e^{-\frac{\pi}{2H_2}} = e^{\frac{\pi}{2H_1}}e^{\frac{\pi}{2H_2}} , \tag{7S}$$

and diving both sides by $e^{\frac{\pi}{2H_1}}e^{\frac{\pi}{2H_2}}$, Eq. (8) of the main text can be recovered, *i. e.*

$$e^{-\frac{\pi}{H_1}} + e^{-\frac{\pi}{H_2}} = 1 , \tag{8}$$

which demonstrates that Eq. (2) is an exact solution for the transcendental equation by van der Pauw (see also lower inset of the Fig 3).

II. Numerical calculations:

The numerical calculations reported in this work were carried out using EXCEL spread sheet and a code in Python. The algorithm to calculate the important parameters of this work starts with the R_2 and R_1 measurements, and the G value estimated from Eq. (12), which is used in the loop to find the first value of $H_1 \approx \pi/8 \sinh(\pi G)$. Comparing $h = H_1/H_2$, calculated using Eq. (10), with $r = R_2/R_1$, determined experimentally, the relative difference $\varepsilon = (h - r)/r$ can be calculated. When $|\varepsilon|$ is smaller than 10^{-6}, the loop is stopped and the parameters L_2/L_1 and H_1 obtained from the loop, and H_2 and $f(L_2/L_1)$ are calculated by Eq. (10) and (11), respectively, and printed out. Furthermore, G and $f(G)$ are calculated directly from Eqs. (13) and (14). The results for some selected R_2/R_1 values are reported in Table 1 of the main text.

Table 1S shows some numerical values for the series $1/H_1$ and $1/H_2$ given by Eqs. (4) and (5), respectively, as a function of L_2/L_1.

Table 1S. Contribution of the first terms (up to $n = 6$) for the series of $1/H_1$ and $1/H_2$ given by Eqs. (4) and (5) for selected values of L_2/L_1. It is also shown values for H_1 and H_2, R_2/R_1, and $f(L_2/L_1)$.

	$L_2/L_1 = 1$	$L_2/L_1 = 2$		$L_2/L_1 = 3$		$L_2/L_1 = 4$	
N	$H_1 = H_2$	H_1	H_2	H_1	H_2	H_1	H_2
0	99.9378	99.9999	98.6046	100.000	96.2483	100.000	94.0337
1	0.0621	0.0001	1.3592		3.4708		5.2082
2			0.0352		0.2560		0.6440
3			0.0011	< 0.0001	0.0225	< 0.0001	0.0956
4	< 0.0001	< 0.0001			0.0022		0.0155
5			< 0.0001		0.0002		0.0026
6					< 0.0001		0.0005
H ($n = 6$)	4.53236	105.120	0.89103	2432.346	0.472219	56280.9	0.320773
$R_2/R_1 = H_1/H_2$	1	1.1799×10^2		5.1525×10^3		1.7552×10^5	
$f(L_2/L_1)$	1	0.389915		0.208336		0.141547	

It is possible to observe that both H_1 and H_2 can be calculated with precision better than 6 digits, using only $n = 6$ in the series. Particular interest is related to the H_1 series, which converges much faster than H_2. This happens because $\sinh(\pi L_2/L_1)$ decreases faster than $\sinh(\pi L_1/L_2)$ for a given $L_2/L_1 \geq 1$. Thus, this work takes advantage of such behavior of $1/H_1$, which can be properly calculated using only the first term of the series ($n = 0$). Furthermore, for the approximated solution given by Eqs. (13) to (15), the truncated function of $1/H_2$ in the first term has also been used. This approximation provides direct calculation of the parameters with error smaller than 1% for the range of experimental interest ($1 \leq R_2/R_1 \leq 10^3$).
